# Guidance on the short hydration method for cisplatin administration

**DOI:** 10.1007/s10147-025-02780-8

**Published:** 2025-06-04

**Authors:** Kiichiro Ninomiya, Kei Kunimasa, Yasuko Kurata, Yuki Sato, Yasuhito Fujisaka, Hitoshi Ishikawa, Katsuyuki Hotta

**Affiliations:** 1 Center for Comprehensive Genomic Medicine, Okayama University Hospital, Okayama, Japan; 2 Department of Thoracic Oncology, Osaka International Cancer Institute, Osaka, Japan; 3 Department of Pharmacy, Okayama University Hospital, Okayama, Japan; 4 Department of Respiratory Medicine, Kobe City Medical Center General Hospital, Kobe, Japan; 5 Department of Medical Oncology, Osaka Medical and Pharmaceutical University, Osaka, Japan; 6 QST Hospital, National Institutes for Quantum Science and Technology, Chiba, Japan; 7 Center for Innovative Clinical Medicine, Okayama University Hospital, 2-5-1, Shikata-Cho, Okayama, 700-8558 Japan

**Keywords:** Cisplatin, Short hydration, Magnesium supplementation, Forced diuretics

## Abstract

Cisplatin is currently used as the central agent in several cancer chemotherapy protocols because of its broad antitumor spectrum and potent antitumor effects; however, preventing cisplatin-induced renal damage and other adverse events is challenging. Recently, several clinical studies have shown that a short hydration method could prevent cisplatin-induced renal damage. In addition, appropriate magnesium supplementation and administration of forced diuretics have been shown to be renoprotective. The Japanese Lung Cancer Society Guidelines Committee has summarized the evidence of renal protection regarding cisplatin administration to provide optimal administration guidance for the cisplatin short hydration method.

## Introduction

Cisplatin is currently being used as a central agent in several cancer chemotherapy protocols due to its broad antitumor spectrum and potent antitumor effects [1, 2]. In 1972, clinical trials of cisplatin were initiated under the direction of the National Cancer Institute; however, its development was temporarily halted due to strong nephrotoxicity. In 1977, it was discovered that its nephrotoxicity could be avoided in animal models by performing massive hydration pre- and post-cisplatin administration and forced diuresis [3]. Another clinical trial reported the safety and efficacy of cisplatin in patients with cancer, and it was approved in the United States in 1978 and in Japan in 1983. However, patients receiving cisplatin at that time had to be hospitalized for hydration using ≥ 2.5–3 L for > 10 h to avoid nephrotoxicity, which degrades the quality of life of patients [4]. Thus, a simple, outpatient-based cisplatin administration system has been long-desired.

Recently, the National Comprehensive Cancer Network (NCCN) chemotherapy order template for cisplatin has been widely used in the US, and several prospective trials of short hydration methods have been reported in Japan. Therefore, summarizing the evidence of short hydration methods utility is clinically significant as it provides guidance for practical hydration methods in Japan; thus, the first edition was published in 2015. Currently, cisplatin is widely administered in Japan using the short hydration method, and the Japanese package insert for cisplatin has been revised to cite this guideline. In the second edition, we evaluated, reviewed the latest evidence, and made revisions that were more relevant to the current clinical practice.

## Cisplatin nephrotoxicity

The mechanism of cisplatin-induced nephrotoxicity is assumed to be as follows: intravenously (IV) administered cisplatin accumulates in the proximal tubules glomerulus post-filtration, damaging the proximal tubular cells [5]. IV administered cisplatin is rapidly bound to proteins and is not filtered by glomeruli; however, some free cisplatin that is not bound to proteins is filtered by glomeruli and eliminated from the body in approximately 2 h [6]. The concentration of free cisplatin peaks at the end of administration and decreases to the measurement limit within 2 h of administration [7]. Therefore, cisplatin-induced acute renal injury is believed to occur approximately 2 h post-administration. Other factors that may contribute to renal injury include a marked decrease in oral rehydration due to gastrointestinal toxicity such as nausea and cisplatin-induced vomiting [8].

## History and changes in cisplatin administration

Although the ideal cisplatin administration method has not yet been determined, several clinical trials have reported that cisplatin can be safely administered. In reports conducted on several carcinomas, predominantly gynecological cancers, cisplatin was safely administered with ≤ 2 L of hydration daily [9, 10]. In a study that compared outpatient and inpatient treatments of patients with cancer receiving chemotherapy with cisplatin for 4–8 h, no difference in treatment efficacy or side effect profile was observed [11]. Furthermore, a randomized trial comparing IV and oral hydration in patients with solid tumors treated with cisplatin found no differences in renal injury incidence [12]. Additionally, in patients with lung cancer or malignant mesothelioma, a short hydration method of approximately 2 L for 4 h was tolerated, with a 4.6% of grade ≥ 2 nephrotoxicity [13]. The US NCCN chemotherapy order template recommends a minimum 1-L and 2-h short hydration regimen for many tumors [14].

However, in Japan, the package insert for cisplatin has long recommended a total of 2.5–5 L of rehydration be administered before, during, and after cisplatin administration over 10 h, and for cisplatin to be administered over 2 h. This information was derived from the clinical trial results and medical environment at the time of approval in 1983. Based on the above hydration method, an increase in creatinine (Cr) levels exceeding grade 2 in Japan has been reported to be 6–7% [15, 16]. Subsequently, the development of new antiemetic agents, such as serotonin antagonists and neurokinin 1 receptor inhibitors, have substantially progressed our understanding of gastrointestinal cisplatin toxicity [17, 18]. Therefore, gastrointestinal toxicity and decreased oral intake, which cause nephrotoxicity, can be avoided.

Two prospective trials conducted in Japan utilized a short hydration method to safely administer cisplatin. The tolerability of the short hydration method (approximately 1.6 L) was evaluated in 44 patients with lung cancer who had adequate renal function and were scheduled to receive cisplatin at ≥ 75 mg/m^2^. At the first cycle’s primary endpoint, using CTCAE v3.0, we observed no grade 2 or worse Cr increase, and only one occurrence (2%) was observed in all cycles [19]. The tolerability of a short hydration regimen (approximately 2.5 L) was also evaluated in 46 patients with lung cancer and adequate renal function who were scheduled to receive cisplatin at ≥ 60 mg/m^2^. No grade 2 or worse Cr increase was observed in the first cycle of the primary endpoint or in any of the cycles [20, 21].

Two prospective trials were conducted to further simplify the hydration method using shorter and lower doses. The tolerability of the short hydration method using approximately 1.7 L was evaluated in 45 patients with lung cancer who had adequate renal function and were scheduled to receive ≥ 60 mg/m^2^ of cisplatin. Using CTCAE v4.0, we detected a case (2%) of grade 2 or worse Cr in the first cycle of the primary endpoint, and no additional cases of worsening were observed in all cycles [22]. The tolerability of approximately 1–1.5 L of short hydration plus oral rehydration was also evaluated in 46 patients with lung cancer and adequate renal function who were scheduled to receive cisplatin at ≥ 60 mg/m^2^. Only one patient (2%) had an increase in Cr level of grade 2 or worse in the first cycle, which was the primary endpoint [23]. These results were highly reproducible among the four independent studies, thus, the short hydration regimen was well-tolerated in all studies (Table 1).

**Table 1:** Four tolerability studies on short hydration methods conducted in Japan

	Ref. [19]	Ref. [20, 21]	Ref. [22]	Ref. [23]
Design	Single arm, prospective tolerability study			
Patient	Lung cancer	Advanced lung cancer	Advanced lung cancer	Lung Cancer
Primary endpoint	Gr 2 or worse Cr increase in CTCAE in the first cycle			
Number	44	46	45	46
Main eligibility criteria				
Age	20–74	≤ 75	≤ 75	20–74
ECOG PS	0 or 1			
Renal function	Serum Cr within the upper limit and Ccr over 60 mL/min			
Dose of cisplatin	≥ 75 mg/m^2^	≥ 60 mg/m^2^	≥ 60 mg/m^2^	≥ 60 mg/m^2^
Median cycle of administration (range)	4 (1 ~ 8)	3 (1 ~ 8)	4 (1 ~ 6)	4 (1 ~ 5)
Cr increase in the first cycle (Gr1 / ≥ Gr2)	NE / 0	4 (9%) / 0	4 (9%) / 1 (2%)	NE / 1 (2%)
Cr increase in all cycles (Gr1 / ≥ Gr2)	7 (16%) / 0	7 (15%) / 0	7 (16%) / 1 (2%)	NE
Unscheduled additional hydration	13 (30%)	10 (22%)	12 (27%)	7 (15%)
Overall response rate	48.0%	31.1%	24%	45.0%

*ECOG* Eastern Cooperative Oncology Group, *PS* Performance Status, *Cr* creatinine, *CTCAE* common terminology criteria for adverse events, *Ccr* creatinine clearance, *Gr* grade, *NE* not evaluable

## Magnesium supplementation

Cisplatin administration induces inappropriate magnesium (Mg) secretion in the proximal tubules and other organs, resulting in hypomagnesemia [24]. Furthermore, in animal models, hypomagnesemia promotes cisplatin reabsorption and increases cisplatin concentration in the proximal tubules, thereby inducing extensive renal injury [25].

Two small randomized controlled trials compared cisplatin administration with or without Mg supplementation to prevent nephrotoxicity in patients with testicular and ovarian cancers, and renoprotective effects were detected in the supplementation group [26, 27]. A systematic review of the renoprotective effect of Mg on cisplatin-induced nephrotoxicity prevention included a meta-analysis of 11 trials that involved cisplatin doses of ≤ 50 mg/m^2^ and reported that Mg supplementation had renoprotective effects [28].

Therefore, Mg supplementation is recommended pre-cisplatin administration; however, the recommended dose and optimal timing of Mg supplementation remain unclear. The NCCN chemotherapy template recommends Mg supplementation at a dose of 8 mEq/L. In several prospective studies in Japan, 8 mEq of Mg was administered pre-cisplatin administration [19, 23] and 4 mEq each pre- and post-cisplatin administration have been used [20, 22].

## Forced diuretics

Both the osmotic diuretic mannitol and loop diuretic furosemide reduce urinary platinum concentrations [29]. Forced diuretics have long been advocated to circumvent cisplatin nephrotoxicity [3]. In particular, a meta-analysis of mannitol, including multiple RCTs and case–control studies, irresepective of the cancer type, showed that mannitol significantly reduced cisplatin nephrotoxicity, especially that of grade ≥ 3 nephrotoxicity events [30].

Two randomized controlled trials of forced diuretics combined with short hydration duration were conducted in Japan (Table 2). A randomized phase II trial compared 60 g of mannitol with 20 mg of furosemide as a forced diuretic in patients aged < 75 years with thoracic malignancies who received cisplatin-based chemotherapy at ≥ 60 mg/m^2^. The primary endpoint of a grade 1 or worse Cr increase in the first cycle was 17.3% in the mannitol group and 24.1% in the furosemide group, wherein non-inferiority of furosemide to mannitol was not demonstrated. Phlebitis was more frequent in the mannitol group (28.8% vs 16.7%) [31]. A randomized phase II trial compared 30 g of mannitol with 20 mg of furosemide in patients with non-small cell lung cancer aged ≤ 75 years who received cisplatin at ≥ 75 mg/m^2^. Since the study was terminated due to slow accrual, making comparisons was difficult. Additionally, the incidence of grade 1 or worse Cr increase was observed in the first cycle18% in mannitol and 9% in furosemide [32].

**Table 2:** Two studies examining forced diuretics in the short hydration method

	Ref. [31]	Ref. [32]
Design	Randomized phase II trial (mannitol vs furosemide)	
Patient	Thoracic malignancies	Advanced NSCLC
Primary endpoint	Gr 1 or worse Cr increase in CTCAE in the first cycle	
Number	106 (52 vs 54)	44 (22 vs 22)
Main eligibility criteria		
Age	20–74	20–75
ECOG PS	0 or 1	
Renal function	Serum Cr under 1.2 mg/dL and Ccr over 60 mL/min	Serum Cr within the upper limit and Ccr over 60 mL/min
Dose of cisplatin	≥ 60 mg/m^2^	≥ 75 mg/m^2^
Cr increase in the first cycle (≥ Gr1)	17.3% vs 24.1%	18% vs 9%
Cr increase in all cycles (≥ Gr1)	23.1% vs 31.5%	27% vs 23%
Unscheduled additional hydration	NE	32% vs 14%

*NSCLC* non-small cell lung cancer, *ECOG* Eastern Cooperative Oncology Group, *PS* Performance Status, *Cr* creatinine, *CTCAE* Common Terminology Criteria for Adverse Events, *Ccr* creatinine clearance, *Gr* grade, *NE* not evaluable

Therefore, as it is unclear which drug should be used as a forced diuretic, each institution should select the drug that they are more familiar with. In four prospective studies conducted in Japan, mannitol was administered in all patients [19–23].

## Practicalities and cautions of the short hydration method

### Selection of patients for whom cisplatin is indicated

First, it is necessary to determine whether the patient is a candidate for cisplatin administration. When using the short hydration method, the following should be addressed.

- Renal function should be well-maintained (e.g., serum Cr level below the upper limit of the facility standard and Cr clearance ≥ 60 mL/min; however, this method may not be accurate in older patients due to the influence of muscle mass, and, if necessary, consider collecting urine over 24-h).
- The patient has a sufficient comprehension of drinking water instructions.
- Cardiac function is preserved (e.g., ejection fraction > 60% on echocardiography, which is expected to tolerate 500 mL/h).
- The patient is in good general condition (ECOG performance status is 0 or 1).

### What to observe during administration

- To confirm the safety of the short hydration method, the decision to introduce it in hospital will be considered on a case-by-case basis.
- Table 3 summarizes the contents that should be included in the supplementation solution. Examples of the administration are listed in Table 4.
- To prevent dehydration, the patients should be encouraged to drink as needed.
- The patient should be informed that drinking large amounts of water may cause hyponatremia via water intoxication, and therefore the patient should not drink excessively.
- The addition of diuretics should be considered as needed, with careful consideration of fluid balance based on body weight changes.
- If patients develop anorexia and have difficulty drinking after receiving cisplatin, additional IV hydration should be aggressively administered to prevent renal failure.
- Patients should be informed that if there is a change in their medical condition, such as continued anorexia, they should contact medical staff immediately.
- Serum Cr level is commonly used to evaluate renal function.
- In nephrotoxicity, the patient should be referred to a nephrologist or other specialist as necessary.

**Table 3:** Performance of the short hydration method

Amount and duration of intravenous rehydration	Mainly saline solution Total 1.5—2.5 L, 3—4 h 30 m
Duration of cisplatin administration	1 h
Oral rehydration	0.5—1 L by the end of cisplatin administration
Magnesium supplementation	Total 8 mEq or more
Forced diuretics	About 150—300 mL of 20% mannitol or furosemide 20 mg

**Table 4:** Example of short hydration method administration

Ref. [19]	Ref. [20]	Ref. [22]	Ref. [23]
Saline solution 50 mL plus palonosetron 0.75 mg plus dexamethasone 9.9 mg (15 m) Anticancer drug plus saline solution (Appropriately) 500 mL of starting solution plus potassium chloride 10 mEq plus magnesium sulfate 8 mEq (1 h) 20% mannitol 200 mL (30 min) Cisplatin plus 250 mL of saline (1 h) 1/4 saline solution 500 mL plus potassium chloride 10 mEq (1 h)	Saline solution 100 mL plus palonosetron 0.75 mg plus dexamethasone 9.9 mg (10 m) Anticancer drug plus saline solution (Appropriately) 500 mL of maintenance solution plus magnesium sulfate 4 mEq (1 h) 20% mannitol 150 mL (15 min) 500 mL total of cisplatin and saline (1 h) 500 mL of saline solution (1 h) 500 mL of maintenance solution plus magnesium sulfate 4 mEq (1 h)	Saline solution 100 mL plus palonosetron 0.75 mg plus dexamethasone 9.9 mg plus magnesium sulfate 4 mEq (15 m) Anticancer drug plus saline solution (Appropriately) 20% mannitol 150 mL (15 m) 500 mL total of cisplatin and saline (1 h) 250 mL of 1/4 saline solution plus potassium chloride 4 mEq plus magnesium sulfate 4 mEq (30 m)	Saline solution 50 mL plus palonosetron 0.75 mg plus dexamethasone 9.9 mg (10 m) Anticancer drug plus saline solution (Appropriately) 500 mL of 1/4 saline solution plus potassium chloride 10 mEq plus magnesium sulfate 8 mEq (1 h) 20% mannitol 200 mL (30 m) Cisplatin plus 250 mL of saline (1 h)
Total 1.6 L, 4 h (Oral rehydration was not specified.)	Total 2.5 L, 4 h 30 m (Include 250 mL of saline for the route, 1 L of oral rehydration recommended.)	Total 1.7 L, 3 h (Include 250 mL of saline for the route, 1 L of oral rehydration recommended.)	Total 1 L or more, 3 h (500 mL of oral rehydration with electrolytes recommended after cisplatin administration.)

Notably, the specifics of the actual use of the cisplatin short hydration method should be discussed with physicians, nurses, pharmacists, and other professionals in advance and share information.

## Conclusions

This review summarizes the evidence and practices of the short hydration method during cisplatin administration, which is becoming increasingly common in daily clinical practice, to further disseminate the short hydration method for cisplatin. Short-term low-dose hydration with Mg or diuretics is recommended for patients in good general condition with adequate renal and cardiac function.
